# What's Killing American Honey Bees?

**DOI:** 10.1371/journal.pbio.0050168

**Published:** 2007-06-12

**Authors:** Benjamin P Oldroyd

## Abstract

American beekeepers reported unusually high rates of colony loss in early 2007 as bees broke from their overwintering clusters. Researchers are struggling to explain what's behind this mysterious disappearance, called colony collapse disorder.

On February 22, 2007, many Americans woke up to media reports that something was awry with their honey bees. A significant proportion of American beekeepers were complaining of unusually high rates of colony loss as their bees broke from their overwintering clusters. Loss of some colonies (say 10%) in early spring is normal and occurs every year. In 2007, however, losses were particularly heavy and widespread—beekeepers in 22 states (including Hawaii) reported the problem. Some beekeepers lost nearly all of their colonies. And the problem is not just in the United States. Many European beekeepers complain of the same problem. Moreover, beekeepers and researchers do not understand the specific causes of the losses.

## Is There a Real Problem?

Were the losses in 2007 within the normal range, or is there something new afoot in the bee industry? If there is something new, what is it? Is it indicative of a general toxic overload of agricultural ecosystems, or a problem confined to the bee industry? Should beekeepers be worried? Should we be worried? The US House Agriculture Committee is sufficiently worried to be holding hearings into the matter, as well they might. Honey bees are essential pollinators: in 2000, the value of American crops pollinated by bees was estimated to be $14.6 billion [1].

**The syndrome is mysterious in that the main symptom is simply a low number of adult bees in the hive. . . There are no bodies, and although there are often many disease organisms present, no outward signs of disease, pests, or parasites exist.**

Here, I try to get to the bottom of the unsolved mystery of colony collapse disorder (CCD)—the official description of a syndrome in which many bee colonies died in the winter and spring of 2006–2007.

## What is CCD?

The syndrome is mysterious in that the main symptom is simply a low number of adult bees in the hive. (This is a bit like going to a previously well-populated hen house and finding hardly any hens.) There are no bodies, and although there are often many disease organisms present, no outward signs of disease, pests, or parasites exist. Often there is still food in the hive, and immature bees (brood) are present. The cause of the loss of bees seems to be the sudden early death, in the field, of large numbers of adult workers [2]. Curiously, the dead colonies tend to be left alone by the two cleptoparasites that normally infest dead honey bee colonies: the wax moth Gallaria mellonella and the small hive beetle Aethina tumida. Could this be due to some toxic residue in the dead colonies? Perhaps this was a contributing factor, but more likely the time of year meant that there were few cleptoparasites about—their abundance is seasonal.

**Figure 1:**
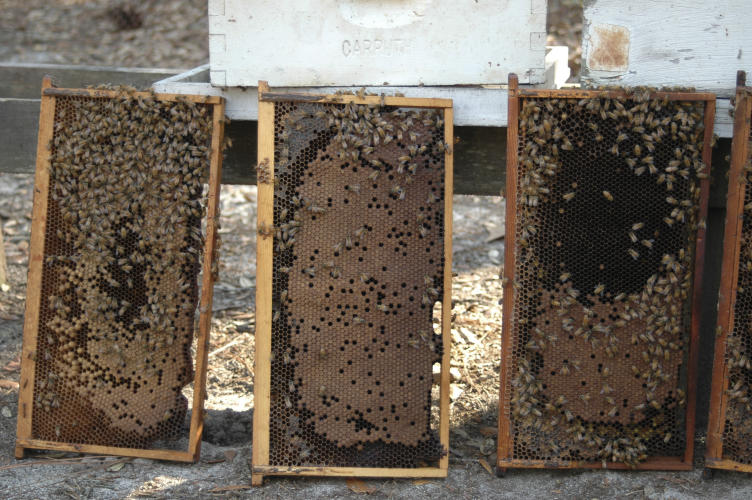
A Colony of Honey Bees Affected by CCD Note the small number of adult workers relative to the large amount of brood. (Photo: Keith Delaplane)

## Were the Losses Unusual?

Some winter losses are normal, and because the proportion of colonies dying varies enormously from year to year, it is difficult to say when a crisis is occurring and when losses are part of the normal continuum. What is clear is that about one year in ten, apiarists suffer unusually heavy colony losses. This has been going on for a long time. In Ireland, there was a “great mortality of bees” in 950, and again in 992 and 1443 [3]. One of the most famous events was in the spring of 1906, when most beekeepers on the Isle of Wight (United Kingdom) lost all of their colonies [4]. American beekeepers also suffer heavy losses periodically. In 1903, in the Cache valley of Utah, 2000 colonies were lost to a mysterious “disappearing disease” following a “hard winter and cold spring” [5]. More recently, there was an incident in 1995 in which Pennsylvania beekeepers lost 53% of colonies [6].

Often terms such as “disappearing disease” or “spring dwindling” are used to describe the syndrome in which large numbers of colonies die in spring due to a lack of adult bees [7,8,9]. However in 2007, some beekeepers experienced 80–100% losses. This is certainly the extreme end of a continuum, so perhaps there is indeed some new factor in play.

## What Are the Possible Causes?

### Diseases and parasites

Honey bees are affected by a large number of parasites and pathogens. Mostly these have a set of well-defined symptoms that do not relate to CCD. For example, there are two major bacterial diseases that affect the brood: European Foul Brood (caused by Mellisococcus pluton [10]), and American Foul Brood (caused by Paenibacillus larvae [11]). There is also a fungal disease of the brood Ascosphaera apis [12]. These organisms have no effect on adult bees but have distinctive symptoms in larvae and pupae.

The parasitic mite Varroa destructor infests brood cells and lives phoretically on adult bees [13]. But heavy mite infections are obvious to professional beekeepers, especially by the stage where colonies are dying of the infestation. So in itself, Varroa infestation is unlikely to cause CCD.

A Tarsonemid mite Acarapis woodi can infest the trachea of adult bees [14] and is now widespread in North America. Acarapis infections were once thought to be the cause of the famous Isle of Wight disease, with symptoms like CCD. However, eminent honey bee pathologist L. Bailey is extremely sceptical that Isle of Wight disease has anything to do with an infectious agent [15]. This is not to say that the Isle of Wight disease is the same as CCD, nor does it exclude the possibility that Acarapis may contribute to CCD.

A protozoan, Nosema apis, infests the guts of adult bees, and when present in high numbers, causes dysentery and early senescence of adult workers [16]. This is also unlikely to be the direct cause of CCD, because the dysentery is obvious and because just about all honey bee colonies are chronically infected with the parasite every spring, even when there are no colony losses. In an interesting twist, however, a new Nosema species, N. cerana, has been recently identified from the Asian hive bee Apis cerana [17] and has now been found on A. mellifera in Europe [18–20]. This “new” pathogen has spread to the US and some researchers speculate that it has contributed to CCD.

More likely to play a role in CCD are a variety of viruses that affect adult bees (Table 1). Most adult honey bees carry symptomless viral infections [21,22]. However, under conditions of stress caused by poor nutrition, inclement weather, or parasitism by V. destructor [23] or N. apis [24], viral populations can increase and cause symptoms in adult bees. The paralysis viruses cause adult bees to tremble and shake, crawling away from the nest unable to fly. Paralysis can certainly reduce the life expectancy of workers dramatically [25], and cause spring dwindling. But in the 2007 outbreak of CCD, there was no evidence of trembling distressed workers. Therefore, the paralysis viruses are not strong candidates for the causative agent of CCD.

**Table 1:**
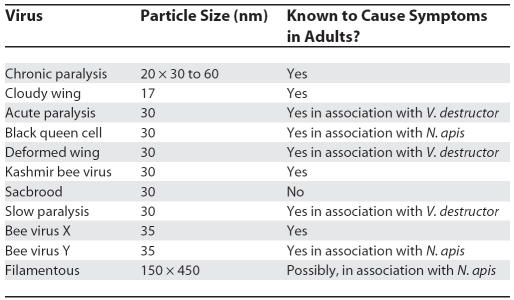
Viruses Isolated from Adult Honey Bees [after 24]

**Anecdotal evidence suggests that CCD is more common in businesses in which bees are trucked large distances and rented for pollination.**

### In-hive chemicals

Like other ranchers, many commercial honey producers are compelled by economic necessity to treat their livestock with a cocktail of drugs and pesticides to keep them healthy. Of particular relevance to CCD are the pesticides used to control the aforementioned brood parasite V. destructor, the cleptoparasitic small hive beetle, A. tumida, and the pest of stored combs, the wax moth G. mellonella. V. destructor was introduced into the US in the late 1980s [13]. It now infests virtually every colony nationwide [41] and has been responsible for the virtual elimination of feral colonies. (Feral colonies are now returning, because the Africanized bee is resistant to the mites [42, 43] and the mite may be losing virulence [41].) However, in the commercial setting, the mites must be controlled—usually chemically.

Apistan, containing the synthetic pyrethroid fluvalinate, is no longer effective for the control of Varroa due to the evolution of resistance [44,45]. It has been replaced with plastic strips containing the organophosphate coumophos [46]. However V. destructor has now developed resistance to coumophos as well [47], and coumophos is now being substituted by Amitraz, a triazapentadiene compound of unknown action. Beekeepers may be increasing dose rates or trying cocktails of chemicals. Some chemicals, particularly fluvalinate, may accumulate in comb wax [48], perhaps exposing commercial honey bees to levels of chemical residue that are inimical to worker longevity. Other beekeepers have tried more “organic” approaches, including fumigation with formic acid [49], oxalic acid, or essential oils [50,51]. Although these approaches do not place insecticides in colonies, they may also be less effective at controlling mites, and can be directly toxic to the bees.

### Agricultural insecticides

American agricultural systems are dependent on the use of pesticides. Where insecticides are used, honey bee losses are common, and where bees are required for pollination, careful management is required to minimize bee losses.

To maintain effectiveness, new insecticides are constantly in development. Sometimes whole new classes of compounds are developed. Before release, all new compounds go through a rigorous registration process that includes assessment of risk to nontarget organisms, including honey bees. Insecticides must be applied in a manner that is nonhazardous to bees and other beneficial organisms. But as with all risk assessment, it is difficult to foresee all possible consequences of wide-spread usage of a particular compound. Perhaps some new insecticide-related phenomenon is now manifesting as CCD.

Bee poisoning is not very likely in early spring in the northern US, where CCD was most widely reported. Moreover, symptoms of acute insecticide poisoning—large numbers of dead and dying bees at the entrance to colonies—are easy to spot. Nonetheless, beekeepers and some scientists remain suspicious that not all new compounds are safe for bees. For instance, wide spread losses of colonies in France in recent years have been blamed on the nicotine-like insecticide Imidacloprid [26]. Imidacloprid acts on the nicotinic acetylcholine receptor of many invertebrates [27]. Because of its low mammalian toxicity, high effectiveness, and high mobility in plant and mammalian tissue, it is often used as systemic insecticide for the control of sap-sucking insects in crops and blood-sucking insects in companion animals [28]. Therein lies the possible problem for honey bees: when applied to plants the insecticide may end up in nectar or pollen.

There is considerable debate about the chances of this happening to a degree that bees are endangered. Some (mainly French) studies report residues of Imidacloprid in nectar and pollen at levels that are potentially dangerous to bees [26,29], while others (mainly North American) detected no residues [30]. Moreover, when Imidacloprid was fed to colonies in syrup or pollen at amounts likely to be found in the field, development and survival of colonies was equivalent in treated and control colonies [31], and contact with the pollen of treated corn plants had no affect on bee longevity [32].

Can we discount the possibility of nicotine-like insecticides as a contributor to CCD? Not completely. When individual bees are exposed to sub-lethal (some would say miniscule) doses of Imidacloprid, their performance in associative learning and memory tests is impaired [33–36]. Perhaps there is a certain level of exposure at which foragers have a higher chance of becoming disorientated and lost.

### Genetically modified crops

Farmers now have access to varieties of such staple crops as corn, cotton, canola, and soybeans, where the genome has been modified to express a bacterium-derived protein with strong insecticidal properties [37]. Crops have also been modified to express herbicide resistance genes, or insect protease inhibitors [37]. Genetically modified (GM) crops offer important environmental benefits in that the need for the application of pesticides on these crops is much reduced. But do the GM crops expressing insecticides in every cell pose a threat to foraging bees? To date, there is no strong evidence that GM crops cause acute toxicity to honey bees [38–40]. Furthermore, the involvement of GM crops in CCD seems less likely when we note that states like Illinois, with huge areas under GM crops, have not reported problems with CCD.

### Changed cultural practices

The honey price is currently depressed. Urbanization and more intensive agricultural practices are reducing honey yields nation wide. These twin factors lead many beekeepers to seek alternative income streams beyond honey production. Chief among these is the leasing of colonies for pollination, particularly almond pollination—a crop that is totally dependent on honey bee pollination. Many crops cause nutritional stress to the bees, or the transport or staging of colonies in holding yards may cause stress. When bees are moved out of these crops, they must feed on high quality pollen to restore body protein levels. This can be achieved by trucking the bees to a location with excellent floral resources or by feeding them. Presumably this is not always done. Anecdotal evidence suggests that CCD is more common in businesses in which bees are trucked large distances and rented for pollination.

Bees also need to feed on high-quality pollen in fall in order to produce long-lived bees that can survive winter [52]. In the US, goldenrod (Solidago virgaurea) is very important in this regard, and the flowering was poor in 2006 in the northeast. Perhaps this contributed to CCD in the following spring.

### Cool brood

Remarkably, honey bees maintain the temperature of their brood nest within ± 0.5 °C of 34.5 °C, despite major fluctuations in ambient temperature [53]. If the brood is incubated a little outside this range, the resulting adults are normal physically, but show deficiencies in learning and memory [54,55]. Workers reared at suboptimal temperatures tend to get lost in the field, and can't perform communication dances effectively [54]. Although entirely a hypothesis, I suspect that if colonies were unable to maintain optimal brood nest temperatures, CCD-like symptoms would be apparent.

## Putting It All Together

We have seen that a large number of factors can produce CCD-like symptoms. We have also seen that CCD is not new: CCD-like symptoms have been known to beekeepers for more than a hundred years but are sufficiently infrequent that when symptoms are severe, beekeepers become concerned that there is something new afflicting their bees.

Clearly CCD is a multifactorial syndrome. Some researchers have suggested that the bees are suffering immunosuppression. Certainly, expression of immune genes in insects is costly [56–58], and if bees are stressed by other causes, they may be less able to mount an effective immune response to pathogens [see Box 1]. This idea is now eminently testable, because the honey bee genome has been sequenced [59], and this provides researchers with new tools to tackle problems like CCD. A microarray of honey bee immune genes and genes from their pathogens is available [60], and this could be used to determine if the known immune genes are underexpressed in colonies suffering from CCD.

**Box 1. Too Narrow a Genetic Base?**

Some researchers are wondering if commercial honey bee stocks are based on too narrow a genetic base—and that this makes them vulnerable to diseases. Honey bee colonies comprise a large number of related animals that live at high densities and exchange food by mouth; these are ideal conditions for the development of epidemics [61]. Workers have numerous defences against disease, including an innate immune system [62] and behaviors in which some workers seek out disease brood and remove it from the colony [63,64]. To be effective, behavioral defences in particular require a high level of genetic variation within colonies. This allows colonies to respond resiliently to the variety of pathogenic and other challenges they face. If all workers are the same, they may solve one problem brilliantly but be more vulnerable to others.

Honey bee queens mate on the wing with 10–30 drones [65], and this is a major means by which they generate genetic variability in their workers [66]. Some scientists have suggested that because Varroa has seriously reduced the number of feral honey bees (see main text), commercial bees are more likely to mate with close relatives than they were in the past, potentially leading to reduced genetic diversity within colonies. Furthermore, imports of honey bees from around the world may mean that commercial honey bees are not well adapted to the local current pathogens and conditions in the US.

**Figure:**
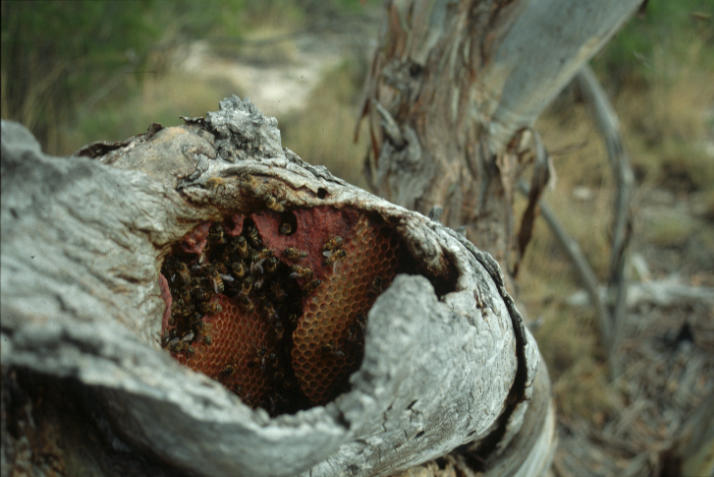
The existence of fewer feral colonies like this one may be lowering the genetic variance in commercial populations. (Photo: B.P. Oldroyd).

I suggest that another possible cause of CCD might simply be inadequate incubation of the brood. Thus any factor—infections, chronic exposure to insecticides, inadequate nutrition, migration in adult population, and inadequate regulation of brood temperature might cause CCD-like symptoms.

My hypothesis could be easily tested by removing brood from several colonies and incubating some of it at optimal temperature and some at suboptimal temperature. The brood would then be used to constitute new colonies in which some colonies comprise workers raised at low temperature and some comprise workers raised at optimal temperature. I predict that the colonies comprising workers reared at suboptimal temperature will show signs of CCD. Moreover, I would not be surprised if they showed higher levels of stress-related viral infections. These effects could act synergistically—more virus leads to shorter-lived, less efficient workers, that in turn leads to suboptimal temperature regulation, and more short-lived bees.

## Abbreviations

CCD: colony collapse disorder
GM: genetically modified
